# A deep learning architecture for leaf water potential prediction in *Populus euramericana* ‘I-214’ from hyperspectral reflectance

**DOI:** 10.3389/fpls.2025.1709473

**Published:** 2026-01-26

**Authors:** Xue-Wei Gong, Qing-Song Yu, Hong-Li Li, Zhuo-Qun Fang, Jia-Xu Guo, Zhao-Kui Li, Heng-Fang Wang, Zhong-Yi Pang, Yan-Hui Peng, Xue-Kai Sun, Guang-You Hao

**Affiliations:** 1 CAS Key Laboratory of Forest Ecology and Silviculture, Institute of Applied Ecology, Chinese Academy of Sciences, Shenyang, Liaoning, China; 2 School of Computer Science, Shenyang Aerospace University, Shenyang, Liaoning, China; 3 Key Laboratory of Oasis Ecology of Ministry of Education, College of Ecology and Environment, Xinjiang University, Urumqi, Xinjiang, China; 4 State-owned Xinmin City Mechanical Forest Farm, Shenyang, Liaoning, China

**Keywords:** conditional generative adversarial network, deep learning, distribution-aware regression, hyperspectral imaging, leaf water potential, smart forestry

## Abstract

**Introduction:**

Leaf water potential (Ψ_leaf_) is a fundamental physiological metric quantifying tree water status and forest drought stress, yet its measurement remains labor-intensive and destructive. Hyperspectral techniques show great promise for retrieving plant physiological traits; however, robust Ψ_leaf_ estimation remains limited by three critical factors: unbalanced data distributions, the need for global–local feature synergy, and inherent uncertainty in point-based regression.

**Methods:**

Here, we propose a deep learning framework (CIDL) that integrates: (1) a conditional generative adversarial network (CGAN) to generate balanced synthetic samples across the full Ψ_leaf_ domain; (2) a feature extractor that combines Inception–ResNet with ACmix (IRAC) to capture local absorption features and long-range spectral dependencies jointly; and (3) a distribution-aware regression network (DARN) to explicitly model the target-variable distribution, thereby enhancing predictive reliability. The model was trained and evaluated using a dataset derived from dehydration experiments on leaves of young *Populus euramericana* ‘I-214’ trees, comprising 229 paired Ψ_leaf_ and hyperspectral reflectance measurements, which were further augmented with 500 CGAN-generated synthetic samples to improve model robustness.

**Results:**

CIDL achieved a prediction accuracy of *R*^2^ = 0.78 and RMSE = 0.27 MPa on the test set, clearly outperforming traditional machine learning methods (mean *R*^2^ = 0.66, mean RMSE = 0.34 MPa) and yielding a modest yet consistent improvement over mainstream deep learning approaches (mean *R*^2^ = 0.76, mean RMSE = 0.28 MPa).

**Discussion:**

These results demonstrate that the proposed CIDL framework provides a generalizable solution for small-sample physiological hyperspectral analysis and offers a reliable, non-destructive pathway for tree water-stress monitoring, with strong potential for applications in smart forestry management.

## 1 Introduction

In recent years, global climate change has intensified, with elevated temperatures and water imbalances leading to frequent droughts worldwide (Trenberth et al., 2014). These climatic shifts pose serious threats to the stability of forest ecosystems (Hammond et al., 2022). Studies have identified drought stress as a principal cause of tree mortality and forest degradation (McDowell et al., 2008; Anderegg et al., 2016). Consequently, early identification and assessment of tree water stress have emerged as key research challenges in the context of global change. Since drought stress initially alters the water status of trees, leaf water potential (Ψ_leaf_), a fundamental physiological metric that quantifies tree water status, has become a critical proxy for predicting tree drought resilience and mortality risk.

Ψ_leaf_ is defined as the potential energy of water in the leaf relative to pure water at atmospheric pressure and temperature, and reflects the dynamic equilibrium between water absorption and transpiration loss (Blackman et al., 2009; Novick et al., 2022). Ψ_leaf_ plays vital roles in regulating hydraulic efficiency, stomatal aperture, and photosynthetic efficiency (Brodribb et al., 2003; Guo et al., 2020). A significant decline in Ψ_leaf_ may induce xylem embolism, obstructing water transport pathways and ultimately leading to plant death (Mantova et al., 2023). Thus, accurate measurement and prediction of Ψ_leaf_ are essential for drought-severity monitoring and ecosystem-stability assessment (Li et al., 2020; Castro-Camus et al., 2013).

However, the generation of Ψ_leaf_ datasets that are both spatially extensive and temporally high-resolution remains technically formidable (Beeri et al., 2018). Conventional techniques such as the Scholander pressure-bomb method and the chilled-mirror dew-point technique (Tyree and Hammel, 1972; Novick et al., 2022), while widely adopted, require destructive sampling and are operationally complex, limiting their application in large-scale, high-frequency monitoring. In contrast, spectral techniques have gained traction due to their non-invasive nature and ability to rapidly extract plant physiological information. Hyperspectral technology, in particular, enables the capture of reflectance information across hundreds of continuous spectral bands (Asaari et al., 2022; Zhang and Zhou, 2018), indirectly revealing leaf physiological status (Gong et al., 2025). Prior studies have shown that Ψ_leaf_ variation induces measurable spectral responses (Tung et al., 2018; Tosin et al., 2021), allowing Ψ_leaf_ estimation through appropriate modeling algorithms.

Researchers have increasingly attempted to apply traditional machine learning algorithms to model plant water-related physiological traits, including leaf water potential. Jin et al. (2017) leveraged visible/near-infrared (Vis/NIR) spectroscopy combined with normalization and selection of 75 sensitive wavelengths to compare the performance of PLS, LSSVR, and RBF NN models in estimating miscanthus leaf water content, demonstrating that the RBF NN based on selected feature wavelengths achieved the highest prediction accuracy ( $R_{p}^{2}=0.9868$, SEP = 0.1536); however, this approach relied on local feature selection and neglected long-range spectral dependencies and imbalanced data distributions. Kovar et al. (2019) employed linear regression and the photochemical reflectance index (PRI) to assess soybean leaf water status, showing that predictions based on visible bands (531 and 570 nm) for equivalent water thickness (EWT) outperformed the traditional 970 nm water absorption band (*R*^2^ = 0.860, RMSE = 0.002), highlighting the limitations of traditional absorption bands and the inability of linear models to capture complex long-range spectral nonlinearities. Cotrozzi et al. (2017) applied partial least squares regression (PLSR) in the SWIR range (1400–2400 nm) to predict oak leaf water potential (*R*^2^ = 0.65), but their linear approach mainly relied on specific water and osmotic substance absorption features, limiting generalization and failing to capture complex nonlinear dependencies. Zhang et al. (2022) demonstrated that PLSR using SWIR bands (1400 and 1900 nm) could accurately estimate leaf water content in broadleaf tree species (*R*^2^ up to 0.98), yet this band combination failed for complex physiological traits such as stomatal conductance, illustrating the limitations of shallow models for nonlinear relationships. Damásio et al. (2023) applied an Extra Trees regression model to predict predawn water potential for five grape cultivars (e.g., Syrah, Touriga Franca) in the Alentejo region, achieving high accuracy (*R*^2^ = 0.833), but the model heavily relied on labor-intensive manual stomatal conductance and meteorological measurements, limiting generalization. Tosin et al. (2021) employed the B-MARS algorithm combined with optimized vegetation indices in the green (520–551 nm) and near-infrared (880–950 nm) ranges to predict grape leaf water potential (RRMSE 13.4%), demonstrating pigment-based proxies; however, this approach relied heavily on site-specific agricultural conditions, restricting transferability. Fishman et al. (2025) applied support vector machines (SVM) to UAV-based hyperspectral data (400–1000 nm) for estimating leaf water potential in mixed forests and identified the red-edge (712 nm) and near-infrared (NIR, 816 nm) regions as most informative. However, their model was highly sensitive to inter-individual variability; reliable accuracy (R² = 0.79) was only achieved after aggregating data to the plot level to mitigate interspecific variation, thereby sacrificing spatial resolution.

In recent years, deep learning methods have achieved significant progress in hyperspectral-based plant physiological parameter estimation. Xiao et al. (2022) combined spectral preprocessing (FD + SNV) with deep transfer learning (fine-tuning CNNs) to enable cross-variety prediction of cotton chlorophyll content (*R*^2^ = 0.87); however, this approach still required labeled target-domain data for fine-tuning and was validated only across limited varieties, with weak generalization. Yue et al. (2023; 2024) pre-trained a VGG network (LACNet) on physically simulated spectra generated by the PROSAIL radiative transfer model to alleviate data scarcity in estimating crop structural and physiological parameters (e.g., LAI and LCC, *R*^2^ = 0.77) and further introduced hyperspectral-to-image transformation (HIT) to adapt one-dimensional spectra to two-dimensional CNNs, enabling soybean chlorophyll estimation (*R*^2^ = 0.78). Nevertheless, these methods rely on mature physical radiative transfer models, limiting their applicability to complex physiological traits such as leaf water potential, which lack well-established models. Zhang et al. (2023) combined near-infrared hyperspectral imaging (900–1700 nm) with a CNN-AT-LSTM-R model to predict rapeseed leaf water content, treating spectral sequences temporally and achieving high accuracy (*R*^2^ = 0.814), but LSTM-based feature extraction focused on local patterns and failed to capture global spectral context.

Despite these advances, the application of deep learning frameworks to leaf water potential (Ψ_leaf_) prediction still faces several critical bottlenecks: (1) Field measurements of Ψ_leaf_ are costly and labor-intensive, especially at low water potentials, leading to limited and highly imbalanced labeled data; under deep learning models that rely on large-scale high-quality annotations, small sample sizes easily cause overfitting and reduce cross-scenario generalization (Xiao et al., 2022). (2) Conventional convolutional architectures, although efficient at local feature extraction (Yue et al., 2023, 2024), are limited by fixed receptive fields and layer-stacking mechanisms, restricting modeling of long-range spectral dependencies and nonlocal structural relationships; for highly continuous, cross-correlated hyperspectral data, such locality bias hinders capturing global spectral context, reducing sensitivity to subtle physiological changes. (3) Moreover, commonly used mean squared error (MSE) loss is highly sensitive to outliers and tends to optimize dominant samples, causing significant bias in sparsely sampled extreme Ψ_leaf_ ranges, making it insufficient for continuous and precise monitoring in complex natural environments (Geng, 2016).

To address these challenges, this study proposes a novel deep learning framework, CIDL (CGAN + IRAC + DARN), which is systematically optimized at three levels: sample augmentation, spectral feature extraction, and imbalanced distribution modeling. First, a conditional generative adversarial network (CGAN) generates spectra conditioned on Ψ_leaf_ labels to enhance data diversity and partially alleviate label imbalance. Secondly, feature extraction employs IRAC (Inception-ResNet with ACmix attention module) to capture both local structural features and global spectral dependencies. Finally, a distribution-aware regression network (DARN) uses adaptive Kullback–Leibler (KL) divergence loss to better model imbalanced label distributions, improving prediction stability and accuracy in rare and extreme Ψ_leaf_ ranges. The proposed model was trained and evaluated using a dataset from dehydration experiments on leaves of a poplar cultivar, consisting of 229 Ψ_leaf_ measurements, and paired hyperspectral images collected under controlled conditions. The objective of this study is to evaluate whether CIDL can provide a reliable solution for hyperspectral-based leaf water potential prediction.

## 2 Materials and methods

### 2.1 Leaf water potential measurement: instrumentation and protocols

**Figure 1:**
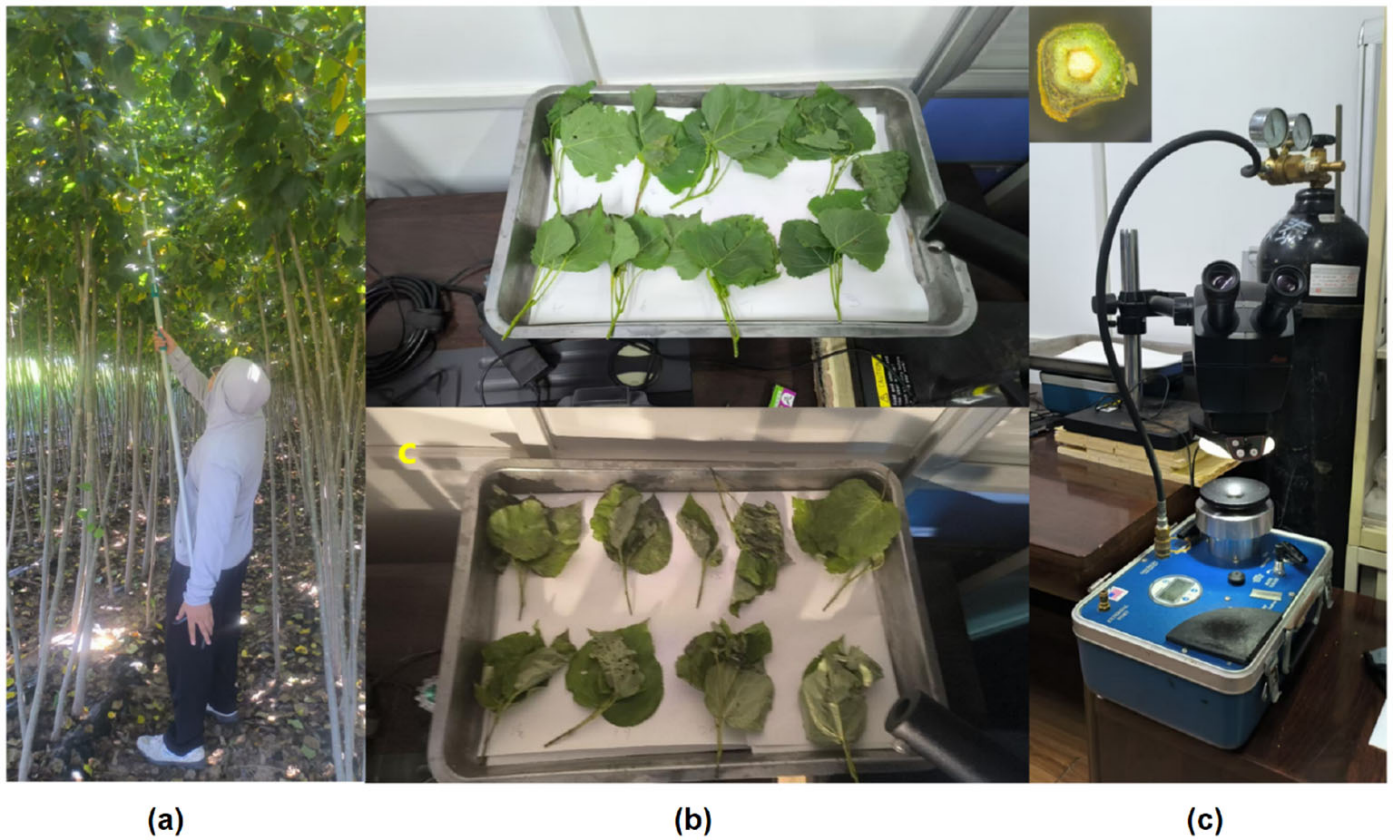
The pressure chamber (PMS 1505D-EXP) used for leaf water potential measurements. **(a)** leaf sampling; **(b)** sample dehydration; **(c)** water potential measurements.

Leaf water potential measurements in this study were conducted on leaves of poplar trees (*Populus euramericana* ‘I-214’). Samples were collected from two-year-old seedlings at the State-Owned Xinmin Mechanical Forest Farm (Yaopu nursery) (**Figure 1a**). Healthy sun-exposed branches were excised at dawn, immediately recut (ca. 3 cm segment removed) under water to allow better rehydration of the branches. With the cutting ends submerged in water and the whole branches covered with opaque plastic bags, the branches were shortly transported to the laboratory. After 2h of rehydration, water potential was measured with a pressure chamber (PMS 1505D-EXP, USA) (Turner, 1981). Key instrument specifications are summarized in **Table 1**. The method involves enclosing the cut branch segment in the chamber, applying pressurized gas, and recording the pressure at which the first droplet of xylem fluid appears; this pressure corresponds to the leaf’s water potential (**Figure 1**) (Roth-Nebelsick et al., 2001).

**Table 1:** Key specifications of the PMS 1505D-EXP pressure chamber used for the measurement of leaf water potential.

Feature	Specification
Maximum Operating Pressure	100 bar
Sample Chamber Material	Solid stainless steel
Sample Chamber Size	Inner dia. 6.35 cm × depth 12.7 cm
Display	Digital pressure gauge (units: bar, MPa, or PSI)
Gauge Range	100 bar
Gauge Accuracy	1 % (over half-scale)
Gauge Diameter	6.35 cm
Overall Dimensions	33 × 28 × 24 cm
Weight	approx. 8 kg
Gas Cylinder	External

Due to the slender petioles of poplar leaves, individual leaf measurements often resulted in air leakage. To increase reliability, we used the terminal twig bearing 3–5 leaves of each sampled branch for water potential measurement. After excision, branches were recut under water, sprayed, and enclosed with black plastic bags to suppress transpiration. This treatment ensured equilibration of water potential, allowing twig measurements to serve as accurate proxies for leaf water potential (Ψ_leaf_) (Mishra et al., 2020).

The Ψ_leaf_ and hyperspectral imagery of the shoot samples were periodically determined during a slow dehydration process (**Figure 1b**). To ensure temporal alignment, immediately before hyperspectral imaging, each shoot was placed in the pressure chamber to measure Ψ_leaf_ (**Figure 1c**). Ψ_leaf_ measurements were taken at intervals ranging from 3 to 20 minutes, shorter during rapid early dehydration and longer during the later stage of dehydration, at a controlled room temperature of about 24°C. A total of 25 shoots were measured, yielding 229 hyperspectral images paired with Ψ_leaf_ measurements. Initial Ψ_leaf_ ranged from -1 to −5 bar (-0.5 MPa), mean values around −10 bar (-1 MPa), and minimum values were as low as −25 bar (-2.5 MPa).

### 2.2 Hyperspectral data acquisition and preprocessing

**Figure 2:**
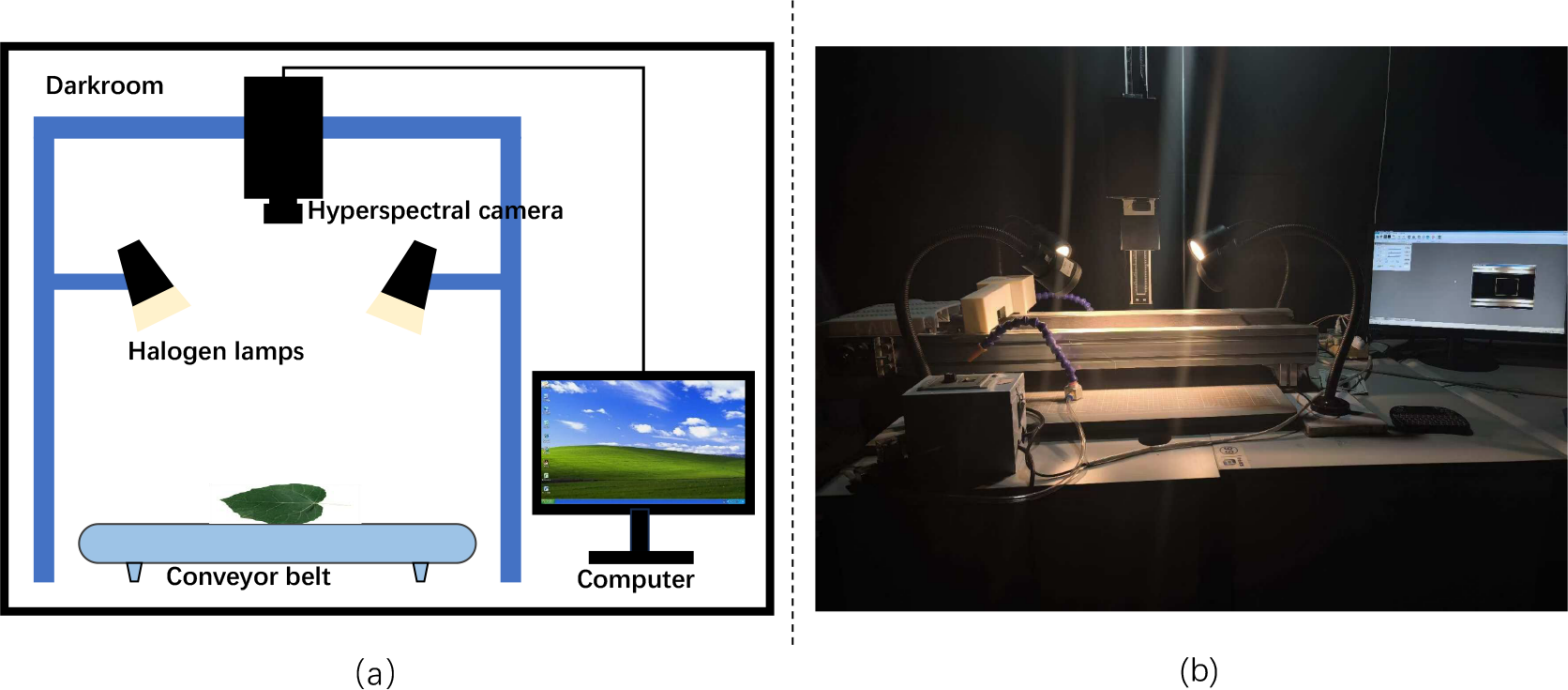
Schematic diagram **(a)** and actual image **(b)** of the hyperspectral image acquisition system built in this study.

The hyperspectral image acquisition system established in this study is illustrated in **Figure 2**. This system is composed of several essential components, including a hyperspectral camera, halogen light sources, a conveyor belt, a standard white reference panel, a camera mount, a display monitor, and a lightproof photographic chamber (Li et al., 2024). The detailed descriptions of these components are provided in the following section.

**Figure 3:**
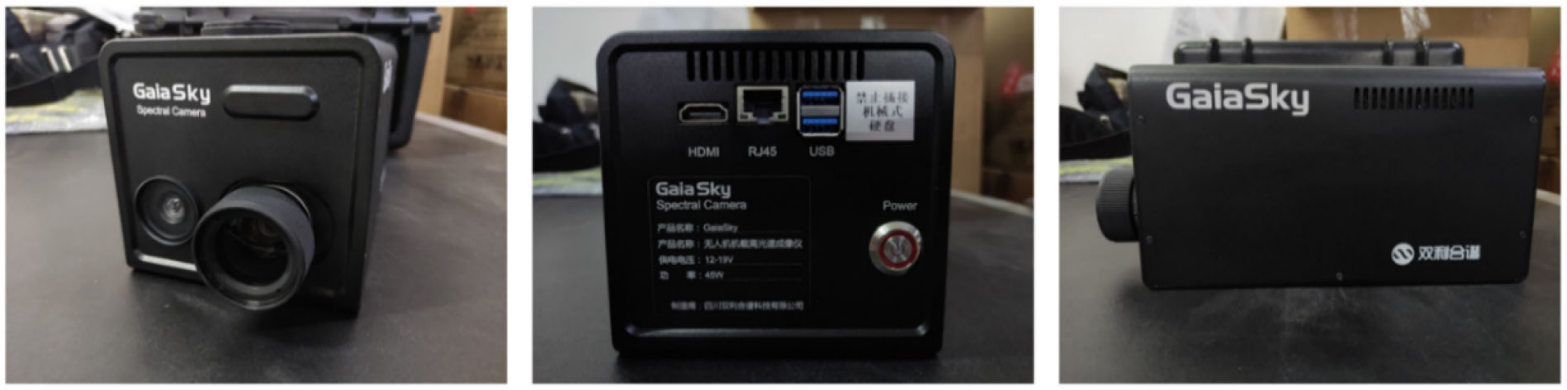
The hyperspectral camera (GaiaSky-mini2) used in this study.

Hyperspectral Camera: In this study, the hyperspectral camera employed is a push-broom hyperspectral imaging spectrometer (GaiaSky-mini2, Sichuan Dualix Spectral Imaging Technology Co., Ltd.), as shown in **Figure 3**. The camera consists of several core components, including an imaging lens, a spectral imager, and an area-array detector. Its detailed specifications and parameters are listed in **Table 2**.

**Table 2:** Key specifications of the GaiaSky-mini2 hyperspectral camera used for the spectral imagery acquisition.

Name	Specification
Spectral range	393.7–1001.4 nm
Spectral resolution	3.5 nm ± 0.5 nm
Sensor	CCD Sony ICX285
Number of bands	176
Full-frame pixels	1057 × 960
Weight	1.0 kg

Halogen Lamps: Halogen lamps are broadband light sources widely used in the visible and near-infrared spectral regions. They are mainly composed of a quartz glass bulb and a tungsten filament, with halogen gas filled inside the bulb. When the filament is heated to a high temperature, it produces a continuous and smooth incandescent spectrum that covers the range from visible to infrared wavelengths without sharp peaks. Due to their relatively low operating voltage and versatility, halogen lamps serve as ideal general-purpose illumination devices (Dotto et al., 2018). In the experiments, four halogen lamps with a power of 50 W each were symmetrically placed around the sample from above to provide the required illumination.

Conveyor Belt System: To ensure uniform illumination and improve the accuracy of image acquisition, this study adopted a push-broom imaging mode in combination with a conveyor belt system to assist the hyperspectral camera in data collection. During acquisition, the sample was placed on the conveyor belt, and a speed controller was used to regulate the belt speed so as to match the scanning speed of the hyperspectral camera. The sample moved at a constant speed along the conveyor belt, while the hyperspectral camera continuously captured images of the designated area during scanning, ensuring the acquisition of high-quality hyperspectral data as the sample passed through the camera lens.

Before collecting spectral data from the samples, a white polytetrafluoroethylene (PTFE) board was placed under the hyperspectral camera to obtain reference images of the white panel, and an opaque lens cap was placed over the lens to acquire dark reference images. These two types of reference images were used to calibrate the spectral reflectance data of the samples collected on the same day. The reflectance calibration formula is expressed as follows:


$$R_{c}=\frac{I_{o}-I_{d}}{I_{w}-I_{d}}\times R_{w}$$

where *R_c_* denotes the calibrated hyperspectral image, *I_o_*, *I_w_*, and *I_d_* represent the original hyperspectral image, white reference image, and dark reference image, respectively, and *R_w_* indicates the reflectance of the white panel (approximately 100%).

### 2.3 Model architecture

**Figure 4:**
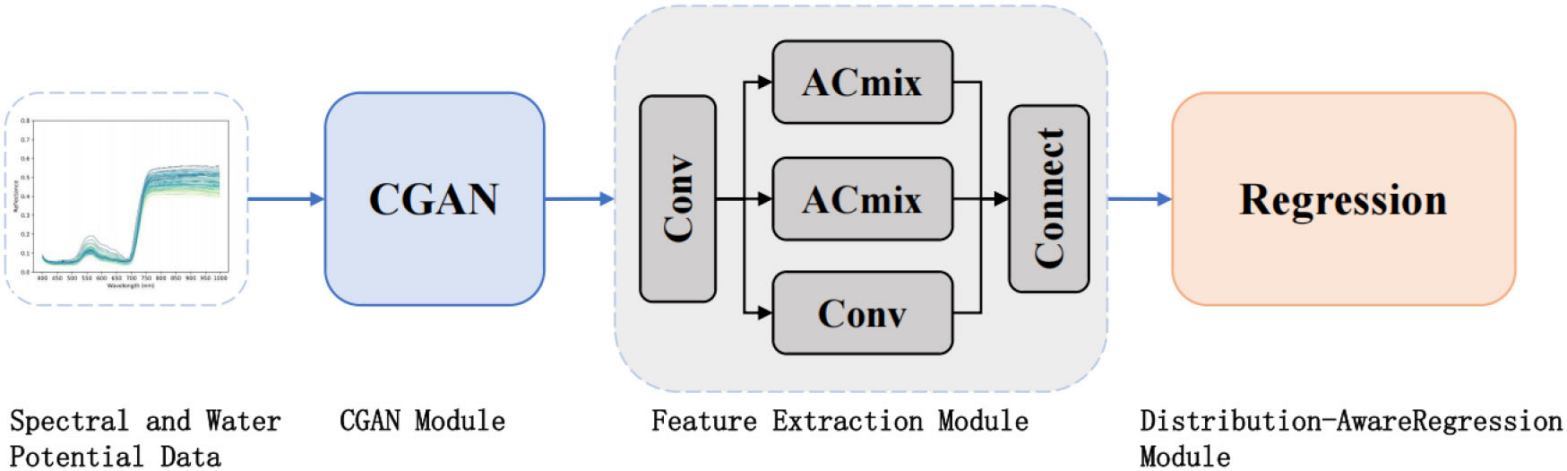
The architecture of the CIDL method.

This study proposes CIDL, a hierarchical deep learning framework integrating Conditional Generative Adversarial Networks (CGAN), Inception–ResNet with ACmix attention (IRAC), and a Distribution-Aware Regression Network (DARN). As illustrated in **Figure 4**, the architecture is composed of three synergistic modules designed to address sample scarcity, feature complexity, and label distribution imbalance, respectively.

First, the CGAN module functions as a conditional data generator. By learning the underlying distribution of real hyperspectral measurements, it synthesizes high-fidelity spectral samples conditioned on Ψ_leaf_ labels. This process augments the training dataset, effectively mitigating the limitations of small sample sizes and enhancing model robustness against noise.

Second, the IRAC module serves as the core feature extractor. Built upon an Inception–ResNet backbone, it incorporates the ACmix attention mechanism. This hybrid design utilizes multi-branch convolutions to capture local spectral variations while simultaneously leveraging self-attention to model global, long-range dependencies across the spectral sequence, thereby maximizing feature representational capability.

Finally, prediction is handled by the DARN module. Unlike traditional point regression, DARN conceptualizes the target variable as a probabilistic distribution (Geng, 2016). By employing a dual-constraint loss function combining Kullback–Leibler (KL) divergence and mean squared error, DARN jointly optimizes distribution matching and expectation calibration. This approach allows the model to quantify uncertainty and improves generalization, particularly when dealing with outliers or noisy labels.

#### 2.3.1 CGAN for hyperspectral data augmentation

In hyperspectral signal analysis, real-world spectral datasets often suffer from limited sample size, imbalanced label distribution, and substantial measurement noise due to the constraints of experimental environments, instrumentation, and sampling costs. These issues can degrade model generalization and reduce regression performance. To address these challenges, we integrate a CGAN (Goodfellow et al., 2014; Mirza and Osindero, 2014) to augment training data, enrich sample diversity, and alleviate noise-induced bias.

CGAN extends the standard GAN framework by conditioning both the generator (*G*) and discriminator (*D*) on auxiliary inputs such as Ψ_leaf_ labels, enabling controlled, context-aware data synthesis. This allows the model to produce synthetic spectra that adhere to specific physiological states, thus improving model robustness and learning stability. Similar strategies have demonstrated effectiveness in hyperspectral contexts, where CGANs successfully model complex spectral distributions and mitigate class imbalance.

**Figure 5:**
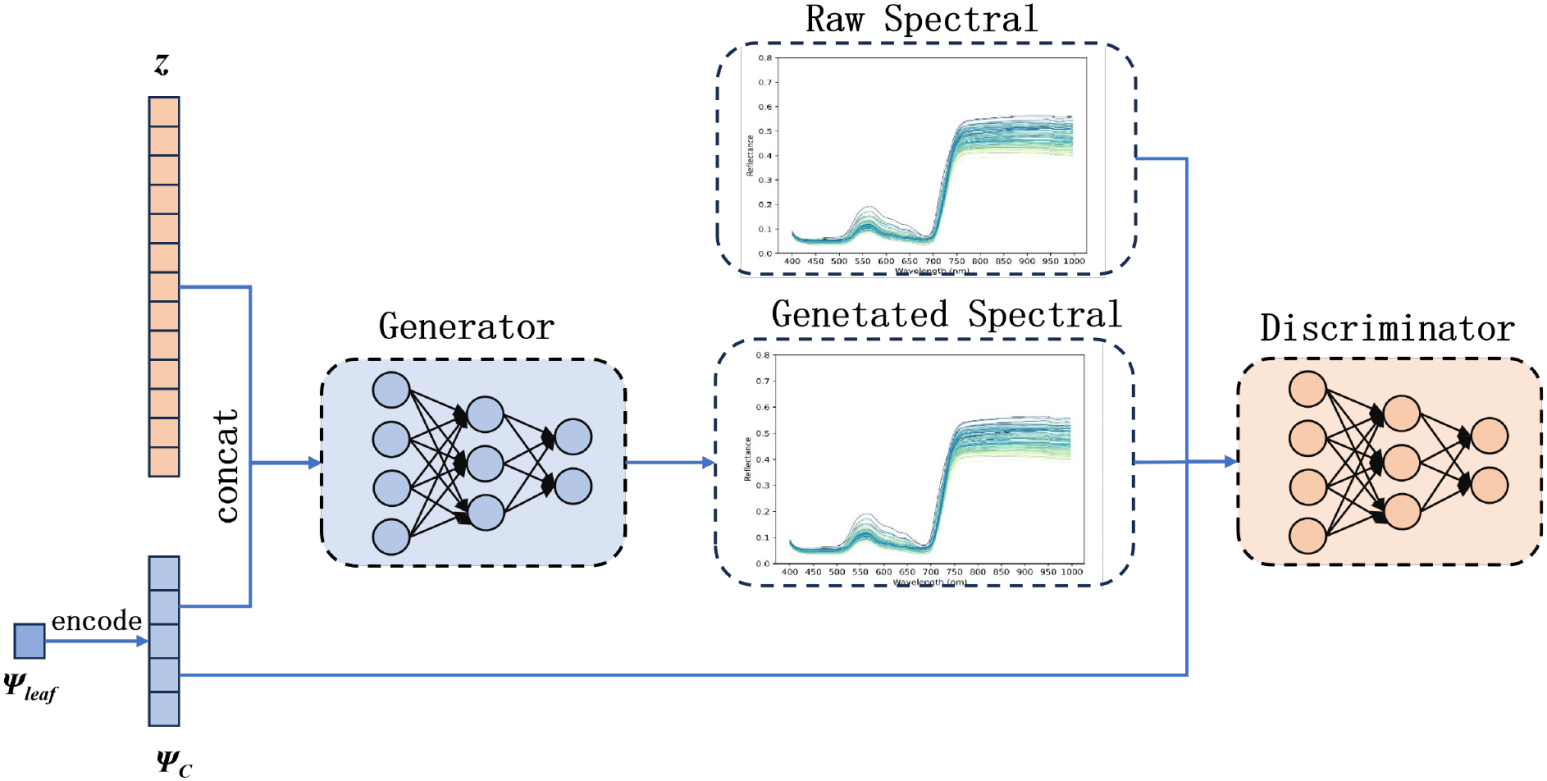
Schematic diagram of the conditional generative adversarial network.

In our CGAN design (see **Figure 5**), the generator *G* takes as input a noise vector *z* and a conditional embedding Ψ*_c_*, which encodes the target leaf water potential Ψ_leaf_. It outputs a synthetic spectrum *G*(*z*,Ψ*_c_*) tailored to that water potential level. The discriminator *D* then evaluates pairs of data—real spectra *x* or generated spectra *G*(*z*,Ψ*_c_*)—along with the same condition Ψ*_c_*, aiming to distinguish authentic from synthetic samples.

Training follows the adversarial min–max game as Equation 1:

(1) $$\begin{array}{l} \underset{G}{\min}\underset{D}{\max}V\left(D,G\right)=E_{x∼p_{\text{data}}\left(x\right)}[\log\;D(x\;|{\text{ Ψ}}_{c})]\\ +E_{z∼p_{z}\left(z\right)}[\text{log }(1-D\left(G(z\;|\;{\text{Ψ}}_{c}\right)\;|{\text{ Ψ}}_{c}))]\; \end{array}$$

Through this adversarial process, the generator becomes proficient at producing high-fidelity, condition-consistent spectra, while the discriminator improves its ability discern measured from synthesized spectra. Importantly, it should be noted that conditional GANs are subject to the well-known issue of mode collapse, where the generator may underutilize the stochastic noise input and produce limited spectral variability under the same conditioning value. Our approach does not rely on a simple regression model but instead captures complex, nonlinear relationships between spectral profiles and physiological states through adversarial learning. Furthermore, ablation studies showed that model performance improved significantly when synthetic data were included (see Section 3.3 below), indicating that the augmented samples contribute meaningful, generalizable information. Unlike conventional augmentation techniques (e.g., adding noise or interpolation), CGAN learns the underlying data distribution and synthesizes spectra that align with experimental observations, conditioned on specific Ψ_leaf_ values (Mao et al., 2019). This yields better control and adaptability across varying physiological conditions. To mitigate the insufficient generalization in sparse data regions, we resampled 500 water potential values in the training set, uniformly distributed across the physiological range of measured Ψ_leaf_. These resampled values supplemented the missing data points in the measured dataset, thereby enriching the diversity of Ψ_leaf_ samples. By inputting these 500 resampled water potential values into the trained generator, we synthesized 500 corresponding spectral curves. This strategy allows the CGAN not only to reconstruct spectral data under different Ψ_leaf_ levels, but also to effectively fill the gaps between actual sampling points. This process alleviates the generalization deficiency in sparse regions and achieves continuous coverage of the spectral-Ψ_leaf_ response relationship. Finally, these synthetic samples were combined with the original training data to form an augmented training set. We evaluated the similarity between real and synthetic spectra using the Spectral Angle Mapper (SAM) metric (Kruse et al., 1993). Specifically, we calculated the SAM value between the mean spectrum of the real samples and the mean spectrum of the CGAN-generated samples. The resulting spectral angle was only 0.0026 rad (0.15°). Considering SAM values below 0.1 rad are considered indicative of high spectral fidelity (Girouard et al., 2004), this confirms that the CGAN-generated spectra closely match the measured data and are of high quality.

#### 2.3.2 IRAC for feature extraction

In this study, we developed a feature extractor named IRAC, which integrates the Inception-ResNet architecture with the ACmix self-attention mechanism (He et al., 2016; Pan et al., 2022). This design aims to extract effective features from input spectral data for the prediction of Ψ_leaf_ values. The feature extractor employs a multi-scale feature extraction approach and optimizes self-attention through the ACmix mechanism, thereby enhancing the model’s ability to capture features from spectral data. The primary task of the feature extractor is to derive key information from the input spectral vectors for subsequent regression tasks. It should be noted that all convolutional and attention modules in the IRAC backbone were specifically redesigned for one-dimensional spectral vectors, rather than directly transferred from image-based 2D architectures, with the aim of jointly capturing local absorption features and long-range wavelength dependencies under a small-sample regime. In its design, this study considers the multi-scale characteristics and sequential dependencies of spectral data. An improved Inception-ResNet (Szegedy et al., 2017) architecture is proposed, incorporating residual connections to ensure effective information transfer. Additionally, a self-attention mechanism is introduced to capture long-range dependencies within the sequential data, further enhancing the feature representation capabilities.

**Figure 6:**
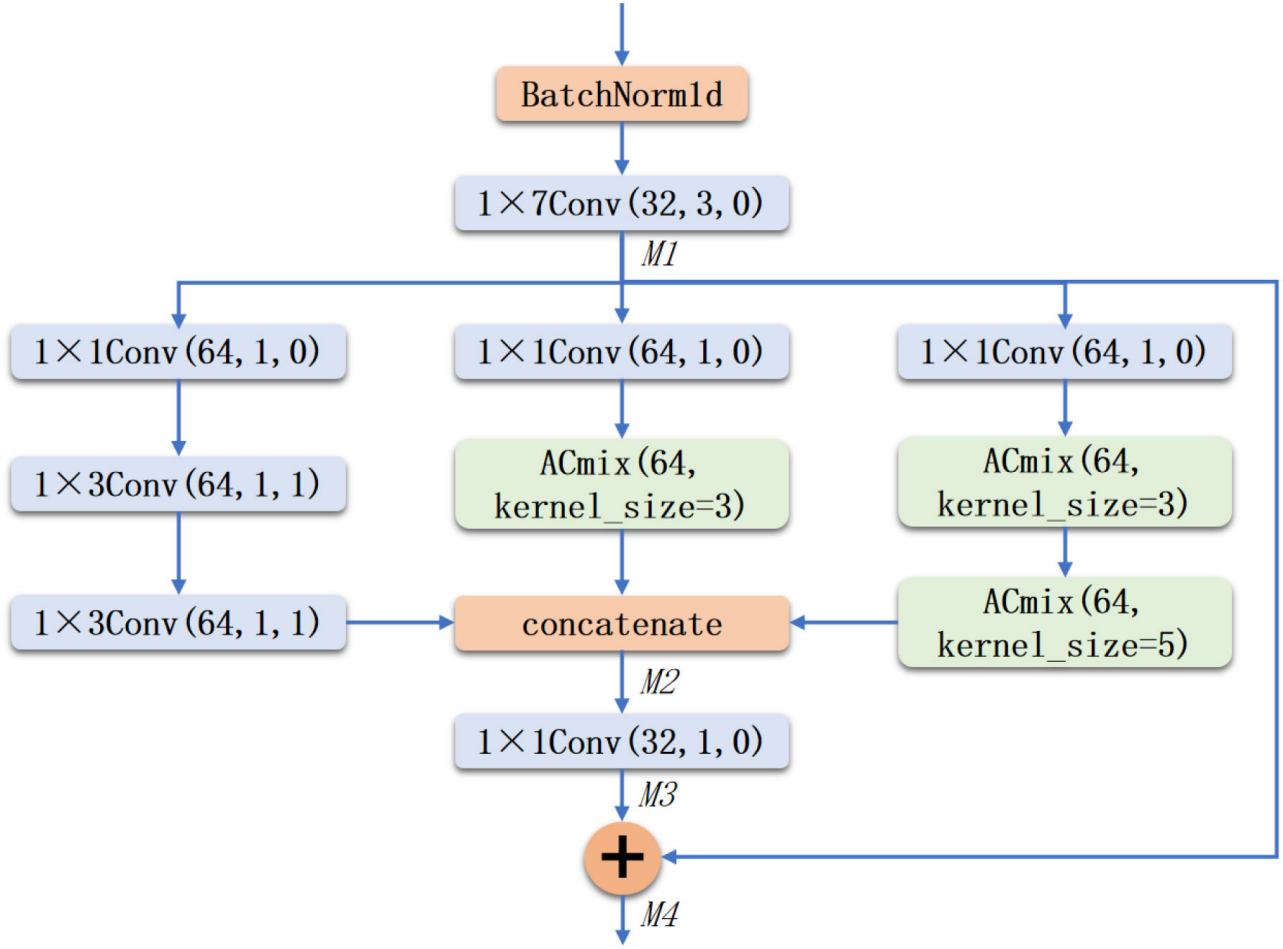
Schematic diagram of the feature extractor.

**Figure 6** illustrates the structural diagram of the feature extractor constructed in this study. Initially, the input data undergoes batch normalization to standardize the data, promoting the stability and efficiency of model training. Subsequently, basic convolution operations generate feature maps, which are then processed through three branches with different convolution kernel sizes, along with residual connections. This design enables the network to capture multi-scale features, thereby augmenting the model’s adaptability to the input data. In these branches, the study selectively replaces basic convolution operations with ACmix modules to enhance the branch’s ability to capture long-distance relationships within the spectral data. The outputs from these three branches are fused along the channel dimension to form a unified feature map. This feature map is then processed through basic convolution operations, and residual connections are applied to combine the features at corresponding positions, producing the final feature map. This step aids in information transmission and retention, reducing information loss during the training of deep networks. The resulting feature map is then flattened and passed to the subsequent module.

**Figure 7:**
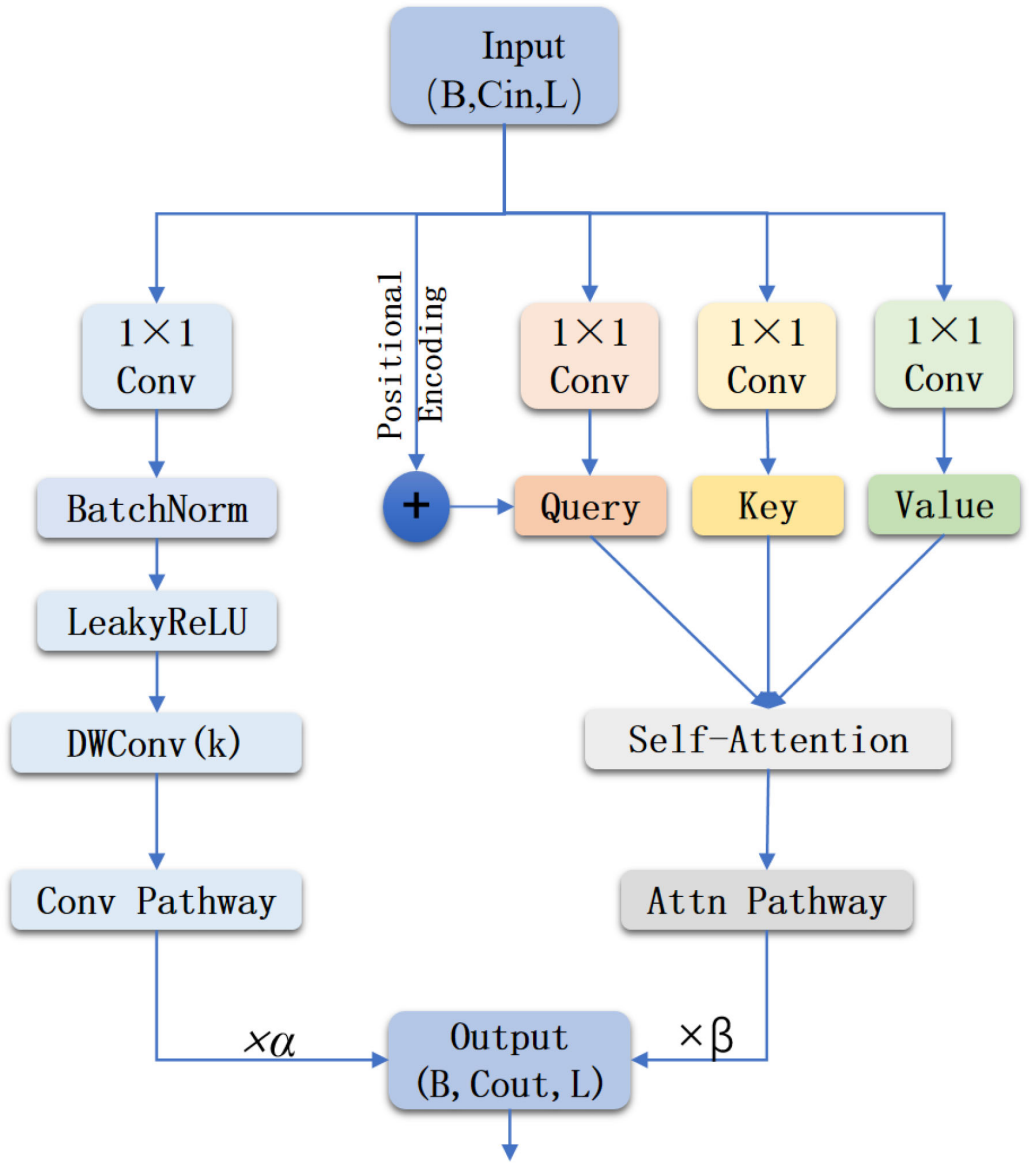
Schematic diagram of the ACmix structure.

A notable characteristic of this feature extractor is its multi-branch structure, where each branch processes the input data using convolution kernels of varying sizes, capturing information at different scales. The outputs from each branch are concatenated along the channel dimension and fused using 1×1 convolutions. To prevent information loss in deep networks, residual connections are introduced in each module, ensuring effective transmission of input information throughout the network and thereby improving training efficiency and network stability. Building upon the previous design, this study introduces the ACmix mechanism to further enhance the network’s expressive capability. ACmix is a hybrid mechanism that combines self-attention and convolution operations. Its core lies in capturing the interdependencies between various positions in the input data through the self-attention mechanism. The structural diagram of the ACmix module is shown in **Figure 7**. In the ACmix module, the input is processed in parallel along two paths: the convolution path extracts local pattern information, while the self-attention path focuses on the relationships between different positions. The convolution path consists of a 1×1 pointwise convolution followed by depthwise separable convolutions, suitable for capturing fine-grained features between adjacent bands. The self-attention mechanism maps the input data into query (Q), key (K), and value (V) vectors, computes their inter-relationships to obtain attention weights, and applies these weights to the value vectors to achieve dynamic feature weighting. Unlike traditional convolution operations that capture only local neighborhood information, the self-attention mechanism adaptively assigns attention weights based on global information, aiding the model in comprehensively understanding the internal structure and semantics of the sequence. In the ACmix module, the self-attention path computes the similarity between queries and keys using dot-product operations, obtaining attention weight distributions, which are then applied to the value vectors to form a fused global feature representation. Additionally, to enhance the model’s sensitivity to sequence order, ACmix introduces a learnable positional encoding module that projects positional information into the attention space, making the model more responsive to position-related features. Subsequently, this feature is fused with the local features extracted by the convolution path to balance local and global modeling capabilities, thereby enhancing the overall expressive power of the model. To merge the outputs from the self-attention and convolution paths, this study employs learnable weighting parameters, denoted as *α* and *β*, to control their influence, allowing for adaptive adjustment under different data patterns. The parameters of the ACmix module include *B* for batch size, *C*_in_ for input channels, *C*_out_ for output channels, and *L* for sequence length. In this study, the input and output channel sizes are set to be equal, facilitating flexible stacking and cascading of this module within the backbone network.

The design of ACmix enables the network to capture both local features and understand global dependencies within the data. The self-attention path dynamically weights the input data by computing interposition relationships, generating richer feature representations. The convolution path is responsible for extracting local region features. By combining the advantages of both, the model can capture information across a broader range of scales. Through residual connections, the outputs from both paths are weighted and fused, further ensuring the transmission and retention of information within the network, thereby preventing potential information loss during training. Through this design, the feature extractor not only extracts effective features from spectral data across multiple scales but also handles long-distance dependencies within the input data, enhancing the model’s learning ability and predictive performance. Ultimately, the feature map is output through weighted fusion and passed to subsequent modules for processing.

#### 2.3.3 DARN for regression optimization

**Figure 8:**
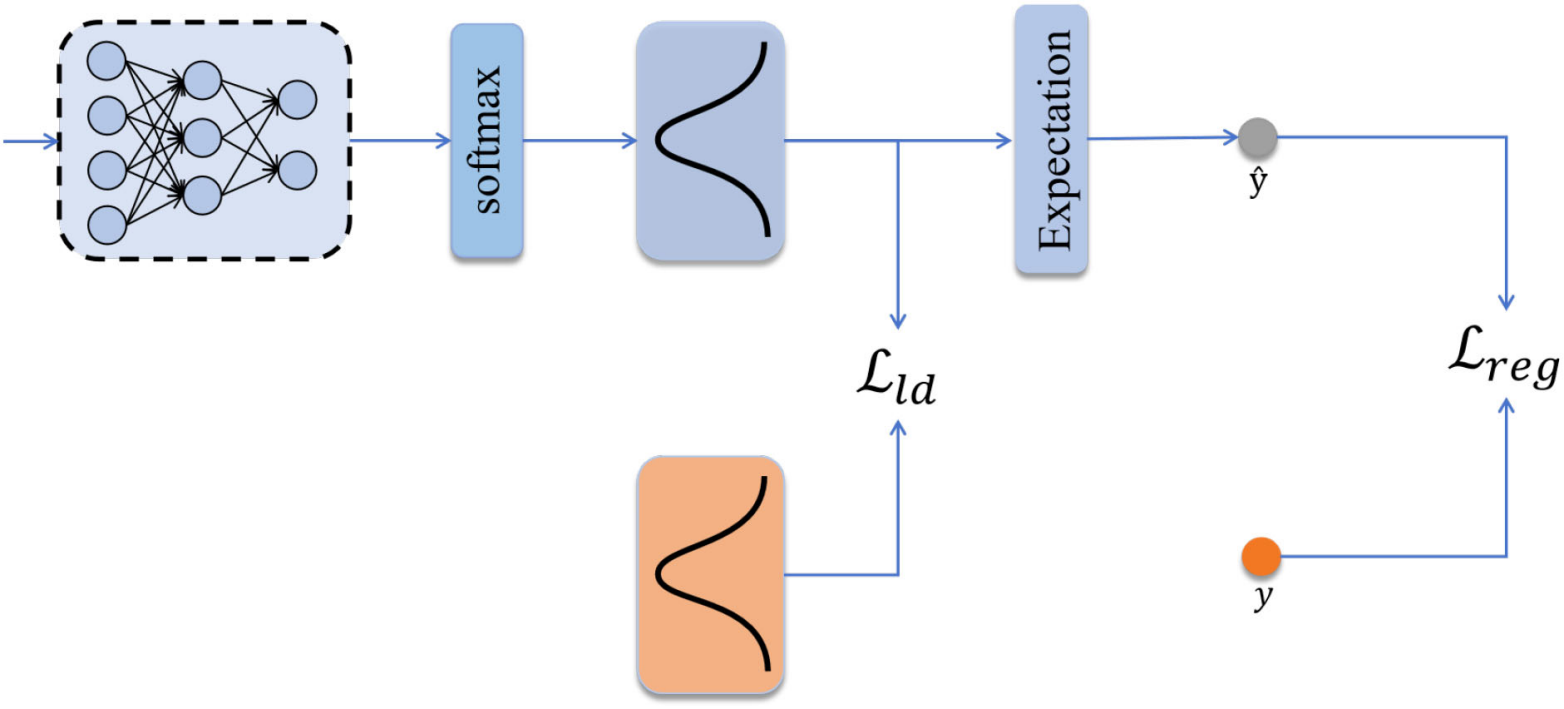
Schematic diagram of the distribution-aware regression network and loss function calculation.

Traditional methods typically employ fully connected regression networks (Regression Network, RN) that directly compute the Mean Squared Error (MSE) loss between predicted and true values. However, MSE loss is sensitive to outliers, which can adversely affect the model’s generalization ability and does not allow for adjustment of the model’s adaptability to data. Therefore, this study introduces label distribution learning and expectation regression on top of the MSE loss to enhance the robustness of the model, as illustrated in **Figure 8**.

Due to gas leakage being prone to occur under high-pressure conditions, which impede the measurement of water potentials in the later stage of the dehydration process, our collected data contain relatively few samples at low (more negative) Ψ_leaf_ values. Consequently, the model fits better within the higher (less negative) Ψ_leaf_ value range. In contrast, under anticipated drought scenarios (where measurements are taken on more water-stressed leaves), the measured Ψ_leaf_ values are expected to fall within the comparatively lower range observed in this study (close to the turgor loss threshold). Therefore, we aim for the model to pay greater attention to this underrepresented but physiologically significant region. Because deep learning models tend to focus on high-frequency data while neglecting low-frequency samples during training, this bias may lead to increased prediction errors in underrepresented regions of the data distribution. To address this issue, we adopt a mixed distribution modeling strategy, which allows the learned distribution to concentrate on the dominant value ranges while simultaneously accounting for low-frequency data. Specifically, at the initialization stage of model training, the label value of each sample is mapped to a probability distribution. The range of water potential values is uniformly partitioned into *k* small intervals of length *l*, where *l* is treated as a hyperparameter. Accordingly, for a sample with a true water potential value *y*, the constructed probability density function is defined as Equation 2:

(2) $$p_{k}=\frac{1}{\sqrt{2\pi \sigma^{2}}}\exp\;\left(-\frac{{\left(l_{k}-y\right)}^{2}}{2\sigma^{2}}\right)$$

Here, *p_k_* denotes the probability that the true water potential takes the value *l_k_*, where *k* represents the number of discretized intervals. Through this formulation, the label value of each sample is transformed into a Gaussian distribution. The parameter *σ*^2^ controls the spread of the normal distribution, ensuring that the distribution remains sufficiently concentrated to capture the main data range while still accounting for tail samples.

In the distribution-aware regression network, the features extracted by the feature extractor are passed through a fully connected network and subsequently normalized using a Softmax function. The normalized outputs are then transformed into a predicted distribution via a probability density function. In this study, the Kullback–Leibler (KL) divergence is employed to quantify the discrepancy between the ground-truth distribution and the predicted distribution. The loss function is defined as Equation 3:

(3) $${ℒ}_{\text{ld}}=\frac{1}{B}\sum_{i=1}^{B}\sum_{k}p_{k}\ln\;\left(\frac{p_{k}}{{\hat{p}}_{k}}\right)$$

By minimizing the loss *L*_ld_, the aim is to align the trend between the true label distribution and the predicted label distribution. To obtain a precise value from the predicted distribution, this study leverages the property of the normal distribution, namely that the expected value of a distribution equals its mean. Accordingly, the leaf water potential is predicted by computing the expectation of the predicted distribution ${\hat{p}}_{k}$. The specific calculation formula is as follows (Equation 4):

(4) $$\hat{y}=\sum_{k}{\hat{p}}_{k}l_{k}$$

By computing a weighted sum of the probabilities of each interval multiplied by the corresponding interval center, the predicted value can be obtained. However, in program implementation, representing the normal distribution with several discrete values does not constitute a truly continuous distribution. As a result, the expected value of the distribution does not exactly match its mean, introducing errors. This directly affects the accuracy of the water potential predicted from the distribution’s expectation. Therefore, to improve regression accuracy, the regression loss is still retained to ensure that the model can predict precise values. Its loss function is defined as follows (Equation 5):

(5) $${ℒ}_{\text{reg}}=\frac{1}{B}\sum_{i=1}^{B}{\left(y_{i}-{\hat{y}}_{i}\right)}^{2}$$

Here, *y_i_* denotes the target value enhanced by the CGAN, and ${\hat{y}}_{i}$ represents the final prediction of the model. *B* indicates the number of samples in a batch. The model minimizes the regression loss *L*_reg_ to reduce the deviation between predictions and ground truth, thereby improving prediction accuracy. Meanwhile, by incorporating the MSE loss and KL divergence, the model emphasizes data imbalance, allowing it to better learn the label distribution and avoid prediction bias caused by imbalanced data. The overall loss function is given as follows (Equation 6):

(6) $${ℒ}_{\text{total}}=C\cdot {ℒ}_{\text{reg}}+{ℒ}_{\text{ld}}+\eta \sum_{i=1}^{n}\theta_{i}^{2}$$

Here, *C* is the coefficient of the regression loss, and *η* is the regularization parameter used to balance the importance of other losses and the regularization term. Ω represents the *L*_2_ regularization term, which helps prevent model overfitting.

### 2.4 Experimental setup

In this experiment, to ensure fairness in hyperparameter selection and reliability in model evaluation, the dataset comprising 229 samples was repeatedly split into 10 independent sets of training (70%), validation (10%), and testing (20%) subsets using 10 different random seeds. The model was trained on each training set and evaluated on the corresponding validation set to identify the hyperparameter combination that achieved the best average performance across the 10 validation rounds. Finally, the model was retrained using this optimal hyperparameter set on each of the 10 combined training and validation sets and evaluated on the corresponding test sets. The final performance metrics were reported as the mean and standard deviation across the 10 test results. The input spectral data were preprocessed using Maximum Absolute Value Normalization. The model was optimized using stochastic gradient descent (SGD) (Peñuelas et al., 1997) with a momentum of 0.9. The batch size was set to 256. To prevent overfitting, the fully connected layers were regularized with a dropout rate of 0.2 within the distributed learning framework. The initial learning rate was set to 0.05, and a learning rate scheduler was employed, reducing the learning rate by a factor of 0.1 after every 100 epochs. The final learning rate was reduced to 1 × 10^−9^. The He initialization method was used for the convolutional layers (He et al., 2015), while the Glorot initialization was applied to the fully connected layers (Glorot and Bengio, 2010).

This study compared the proposed method with traditional approaches (PLSR and SVM) and deep learning approaches, including SpectraNet32, DeepSpectra, CNN, and Transformer (Zhang et al., 2019; Alzubaidi et al., 2021; Gao et al., 2025). We employed repeated hold-out validation with 10 iterations to determine the optimal number of latent variables, with the maximum set to 40. Standardization was applied to the data before prediction. It is important to note that due to the differences in datasets and spectral quantities, these comparison algorithms cannot be directly applied to the data collected in this study. Therefore, hyperparameter optimization must be performed. Specifically, the batch size was uniformly set to 256, with learning rates chosen from {0.01,0.05,0.1}, and regularization coefficients selected from {0,0.00001,0.000001}. The number of epochs was chosen from {100,200,300}.

The neural network models in this study were implemented using the PyTorch Lightning 1.8.3 framework in a Python 3.9.7 environment. All experiments were conducted on high-performance hardware, specifically the Precision 7920 Tower workstation equipped with an Intel(R) Xeon(R) Gold 6226R CPU, 2.9GHz, 128GB of system memory. The graphics processing unit (GPU) was an NVIDIA GeForce RTX 3090 with 24GB of memory, ensuring efficient computation and large-scale data processing capabilities. The computational efficiency in terms of training and testing time for the proposed CIDL method and the baseline approach is presented in **Supplementary Table S1**.

## 3 Results and discussion

### 3.1 Analysis of spectral features

**Figure 9:**
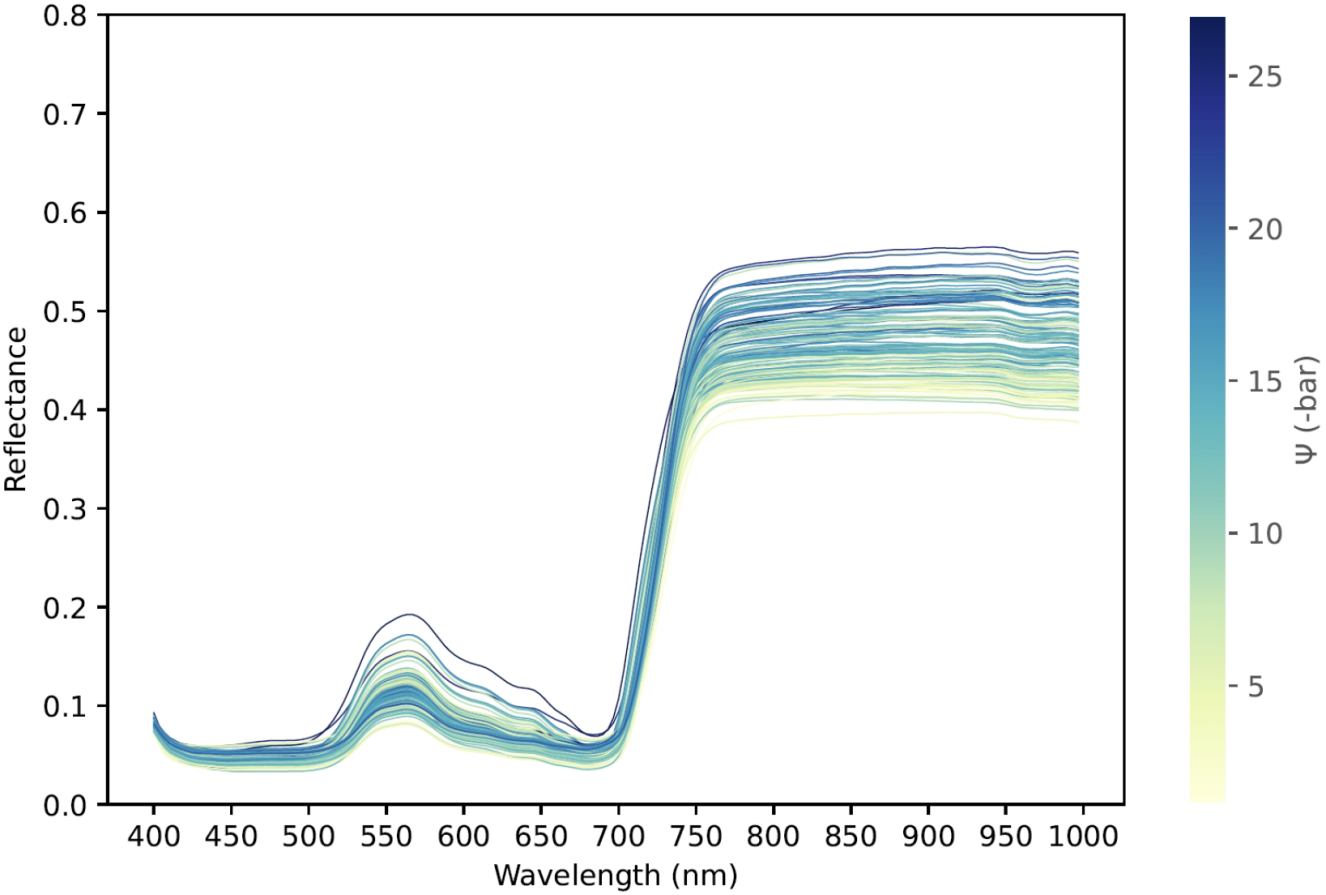
Spectral reflectance of *Populus euramericana* ‘I-214’ leaves with contrasting leaf water potential (Ψ_leaf_). Average hyperspectral curves from poplar leaves are colored by their measured Ψ_leaf_ values.

This study acquired 229 leaf spectral images together with their corresponding Ψ_leaf_, covering wavelengths from 393.7nm to 1001.4nm, across a total of 176 bands. Thus, the dataset spans both the visible region (VIS, 400nm–700nm) and the near-infrared region (NIR, 700nm–1000nm), as shown in **Figure 9**. The spectral curves exhibit low reflectance in the visible region and gradually increase in the near-infrared region, presenting the characteristic spectral signature of vegetation.

In the wavelength range of 400–500nm, the reflectance is relatively low, likely due to chlorophyll absorption. A pronounced red-light absorption band is observed near 680nm. Beyond 700nm, reflectance increases sharply as the spectrum enters the high-reflectance near-infrared region, indicating enhanced scattering by internal cellular structures. Additionally, after approximately 900nm, the reflectance curve tends to plateau, which may be influenced by water content or cellular architecture (Li et al., 2009; Cai et al., 2008).

It is noteworthy that the spectral curves exhibit a correlation with Ψ_leaf_. Overall, samples with lower Ψ_leaf_ tend to display higher reflectance, especially in water-sensitive near-infrared regions, whereas those with higher Ψ_leaf_ exhibit relatively lower reflectance. This trend may be attributed to the absorptive and scattering properties of water, particularly in the near-infrared region, where water absorption is strong; hence, leaves with more negative water potential tend to reflect more in this region.

### 3.2 Model comparison and performance evaluation

This study proposes a deep-learning method for spectral data, named CIDL. To validate its effectiveness, CIDL was compared against several traditional and deep learning approaches—PLSR, SVR, SpectraNet32, DeepSpectra, CNN, Transformer—evaluated by training-set determination coefficient $\left(R_{\text{Train}}^{2}\right)$, root mean square error of calibration (RMSEC), test-set determination coefficient $\left(R_{\text{Test}}^{2}\right)$, root mean square error of prediction (RMSEP), standard deviation ratio (SDR), etc. The experimental results are summarized in **Table 3**. From the test-set determination coefficient $R_{\text{Test}}^{2}$, CIDL achieved 0.7842 ± 0.0448, outperforming all comparison methods, which demonstrates its superior generalization ability. Traditional methods PLSR (0.7310 ± 0.0716) and SVR (0.5914 ± 0.0872) performed relatively modestly due to insufficient extraction of complex spectral features. Among deep learning baselines, CNN (0.7720 ± 0.0487) and SpectraNet32 (0.7793 ± 0.0525) surpassed traditional approaches but still slightly lagged behind CIDL.

**Table 3:** Performance comparison of CIDL against benchmark and deep learning models for leaf water potential prediction. Test *R*^2^, coefficient of determination on the test set; RMSEP, root mean square error of prediction; SDR, standard deviation ratio; MAE, mean absolute error; RPD, ratio of prediction deviation; and RPIQ, ratio of performance to interquartile distance.

Model	Train *R*^2^	RMSEC (bar)	Test *R*^2^	RMSEP (bar)	SDR	MAE (bar)	RPD	RPIQ
PLSR	0.9142	1.6818	0.7310	3.0019	1.9919	2.3794	1.9968	3.0414
SVR	0.9229	1.5916	0.5914	3.7099	1.6130	2.7692	1.6130	2.4697
SpectraNet32	0.8548	1.3344	0.7793	2.7249	1.9272	2.2178	2.1937	3.3527
DeepSpectra	0.7741	2.4835	0.7520	2.9060	2.0297	2.3015	2.0526	3.1410
CNN	0.8594	1.4316	0.7720	2.7717	2.1285	2.1680	2.1526	3.2887
Transformer	0.7040	3.1040	0.7448	2.9362	2.0063	2.3247	2.0290	3.0814
CIDL	0.9867	0.8352	0.7842	2.7095	2.4965	2.0848	2.2108	3.3835

Regarding RMSEP, CIDL yielded the lowest predictive error at 2.7095 ± 0.3745 bar, outperforming all alternatives. Traditional methods, e.g., PLSR (3.0019 ± 0.3699 bar) and SVR (3.7099 ± 0.4281 bar), exhibited higher errors, underscoring their limitations in modeling spectral data. Among deep learning methods, DeepSpectra (2.9060 ± 0.3481 bar) and Transfomers (2.9362 ± 0.2695 bar) achieved reduced errors via complex architectures and augmentation strategies, but lagged behind CIDL.

From the standard deviation ratio (SDR), CIDL achieved a value of 2.4965 ± 1.1120, significantly surpassing all comparative methods, which demonstrates its superior spectral modeling capability and stability. In contrast, traditional methods PLSR (1.9919 ± 0.2670) and SVR (1.6130 ± 0.2121) yielded relatively lower SDR, reflecting their limited ability to capture complex spectral patterns. Among deep learning approaches, DeepSpectra (2.0297 ± 0.1803) and CNN (2.1285 ± 0.2385) improved SDR to some extent by combining deep feature extraction, yet still fell short of CIDL, indicating CIDL’s greater stability in modeling and feature representation. Consistently, among all compared models, our CIDL method achieved the lowest MAE, as well as the highest RPD and RPIQ values (**Table 3**).

**Figure 10:**
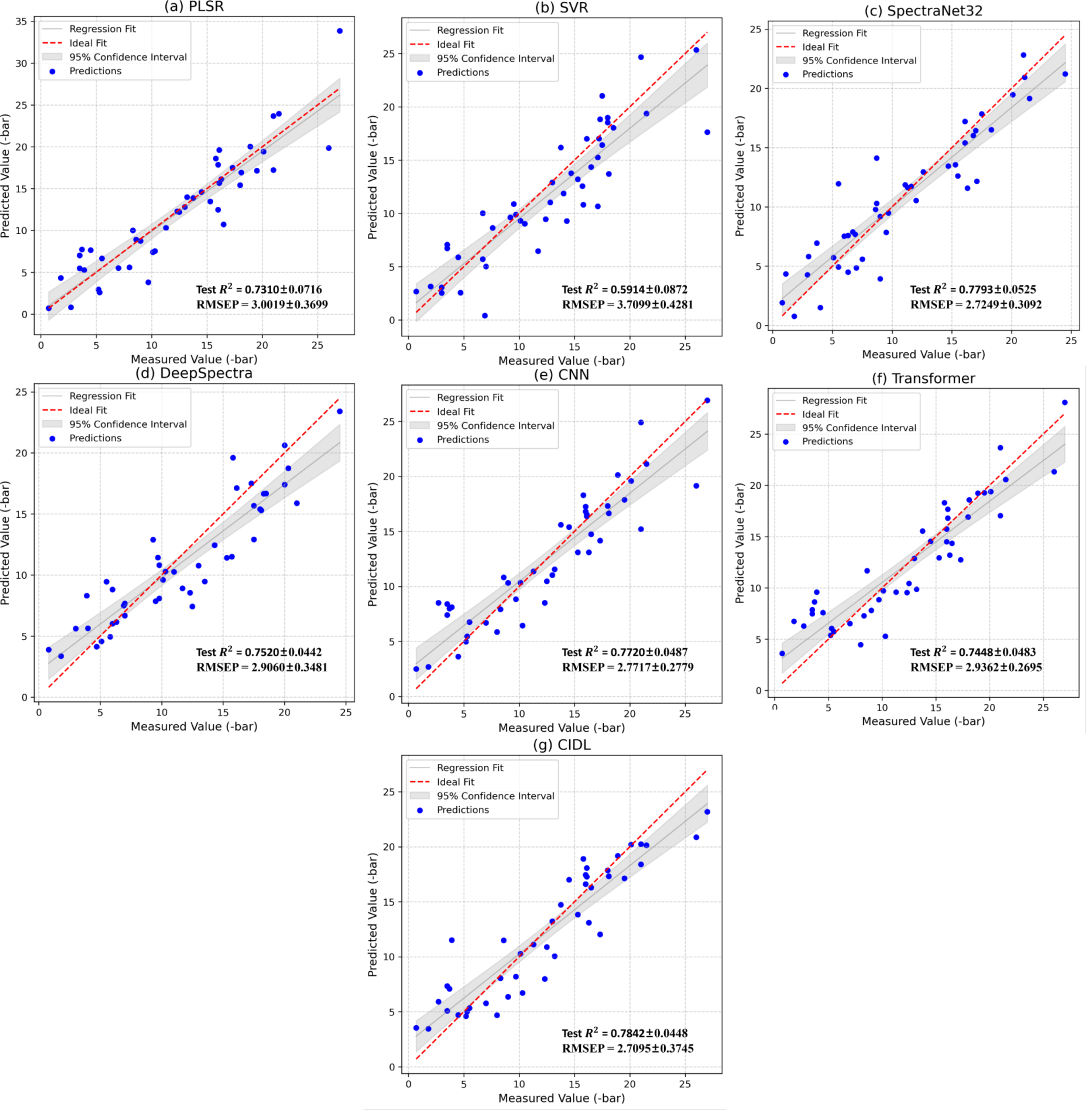
Measured vs. model-predicted scatter plots for leaf water potential (Ψ_leaf_) of *Populus euramericana* ‘I-214’ in the test set. PLSR, a; SVR, b; SpectraNet32, c; Deepspectra, d; CNN, e; Transformer, f; CIDL, g. In each panel, the horizontal axis denotes measured Ψ_leaf_ and the vertical axis denotes predicted Ψ_leaf_. A black dashed line represents the ideal prediction line (y=x), while blue dots indicate individual sample measurements and predictions.

From the scatter plots of predicted versus measured values on the test set, it is evident that the data points from the CIDL model are tightly clustered around the central diagonal and exhibit a closer alignment compared to other methods, underscoring its superior predictive performance (**Figure 10**).

Taken together, CIDL demonstrates excellent performance in spectral regression tasks, particularly excelling in $R_{\text{Test}}^{2}$ and SDR (**Supplementary Table S2**). Compared to traditional and other deep learning approaches, CIDL maintains high predictive accuracy while reducing error and enhancing stability. These findings suggest that the proposed method significantly enhances the accuracy of spectral data modeling and offers a promising direction for future hyperspectral analysis.

### 3.3 Contribution of model components to predictive performance

To comprehensively assess the contribution of each component in the proposed CIDL method, we conducted a series of ablation experiments. All experiments are based on an Inception–ResNet feature extractor and a fully connected regression network (denoted as the *Baseline*). We then incrementally introduced the CGAN module, the ACmix module, and the DARN module to evaluate their individual impacts on model performance. The detailed results of the ablation study are presented in **Table 4**.

**Table 4:** Ablation study on the contribution of individual components to the CIDL framework.

Configuration	Test $R^{2}$
Baseline	0.7286±0.0585
Baseline + CGAN	0.7409±0.0477
Baseline + ACmix	0.7097±0.0476
Baseline + DARN	0.7338±0.0360
Baseline + CGAN + ACmix	0.7443±0.0417
Baseline + CGAN + DARN	0.7791±0.0567
Baseline + ACmix + DARN	0.7479±0.0396
Baseline + CGAN + ACmix + DARN (CIDL)	0.7842±0.0448

Results show that using only the Inception–ResNet feature extractor and the fully connected regression network (Baseline) yields a test set performance of 0.7286 ± 0.0585 $\left(R_{\text{Test}}^{2}\right)$. This indicates that, while the Inception–ResNet architecture is capable of effectively extracting spectral features and providing a certain level of regression performance, there remains considerable room for improvement. By introducing the CGAN module on top of the Baseline, the test performance increases to 0.7409 ± 0.0477 $\;\left(R_{\text{Test}}^{2}\right)$, corresponding to an approximate 1.7% improvement. The addition of CGAN effectively augments the training data, enhances data diversity, and mitigates overfitting, thereby boosting regression accuracy. These results demonstrate that the CGAN-generated spectral data partially compensate for the insufficiency of the original dataset and enable the model to learn more generalizable features.

With the Baseline and CGAN in place, the subsequent inclusion of the ACmix module further elevates the test performance to 0.7443 ± 0.0417 $\left(R_{\text{Test}}^{2}\right)$. The ACmix module integrates convolutional operations with self-attention mechanisms, thereby more effectively modeling both local and global spectral features and enhancing the extraction of key wavelength information. Consequently, this result indicates that ACmix contributes to a stronger spectral feature representation and further improves the model’s predictive performance.

Finally, upon integrating the DARN into the preceding three constituent modules, the overall test performance ascends to an even higher plateau of 0.7842 ± 0.0448 $\;\left(R_{\text{Test}}^{2}\right)$, translating into an additional improvement of approximately 5.9%. By explicitly encoding and leveraging the intrinsic distributional nuances embedded within the target labels, DARN endows the model with an augmented capacity for discerning subtle yet critical temporal and magnitude trends inherent to Ψ_leaf_, thereby eliciting a pronounced enhancement in predictive fidelity across regression tasks.

The ablation results summarized in **Table 4** indicate that each component of the CIDL framework contributes positively to model performance. Specifically, incorporating CGAN offers the largest increase, validating its effectiveness as a data augmentation strategy in spectral regression. The ACmix module significantly enhances spectral feature extraction, yielding additional gains, while DARN further refines model prediction. Ultimately, the full CIDL model achieves a test-set *R*^2^ of 0.7842 ± 0.0448, approximately 7.6% higher than the Baseline. These results confirm that our proposed method substantially improves both generalization capability and regression accuracy in spectral data analysis.

### 3.4 Sensitivity analysis and hyperparameter evaluation

**Figure 11:**
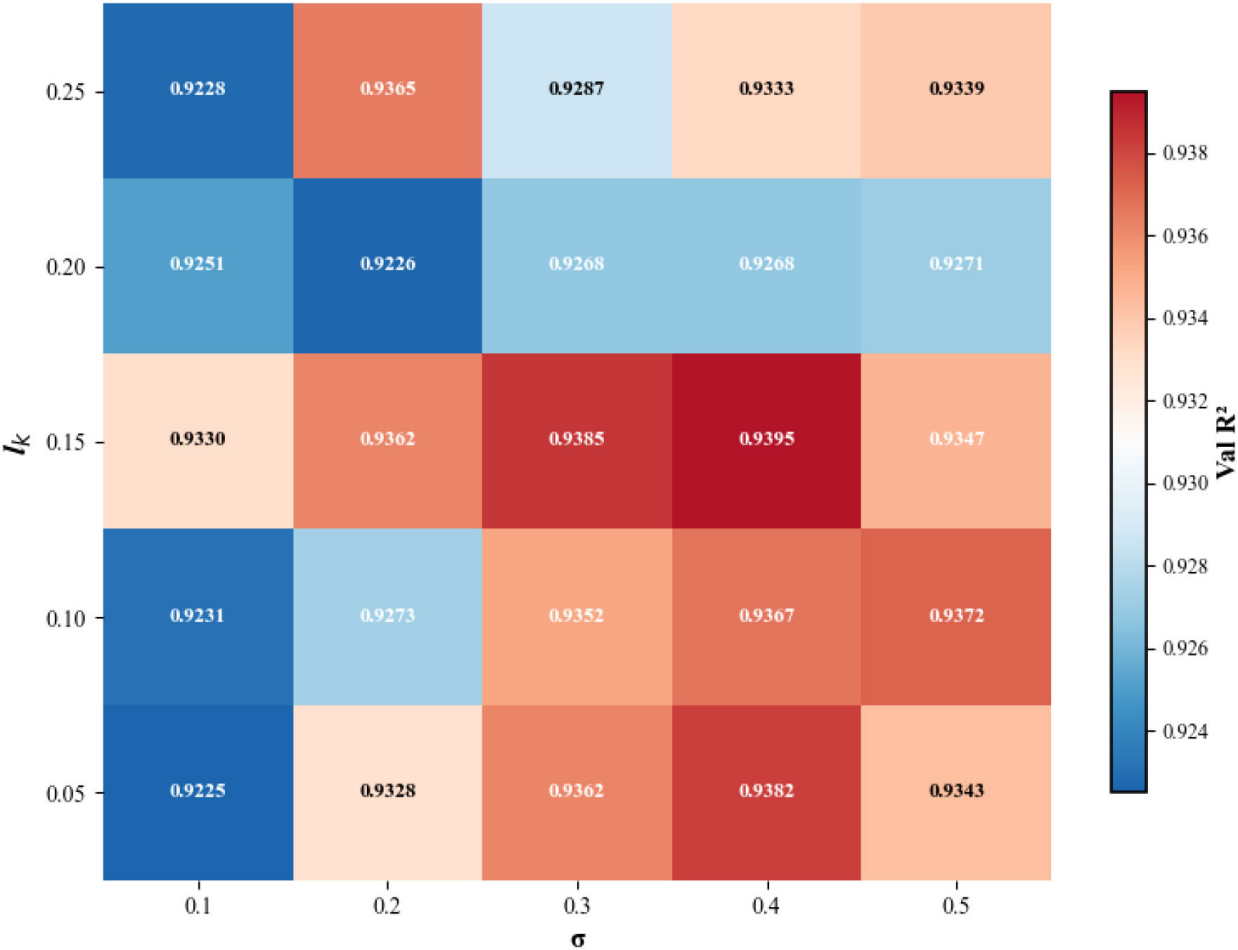
The performance (validation set R²) of the CIDL model across different combinations of the standard deviation (*σ*) and the interval length (*l*). The color intensity corresponds to the R² value, with redder shades indicating higher predictive accuracy. The optimal performance (R² = 0.9395) was achieved with the hyperparameter set *σ* = 0.4 and *l* = 0.15.

To optimize the performance of the predictive model, three important hyperparameters were introduced during the model training process: the standard deviation *σ* of the distribution of the regression network, the interval length *l* of the label distribution, and the constant *C* in the calculation of the linear shrinkage weight. The hyperparameter *σ* determines the smoothness of the label distribution. The interval length *l* defines the size of the region where the label values are distributed. A smaller *l* value provides a more detailed estimate of Ψ_leaf_, but it also increases the complexity of the model training. The hyperparameter *C* affects the generation of the weight *w_e_* in the KL divergence calculation, which in turn influences the impact of low-frequency data on the calculation process. To determine the optimal values of *σ* and *l*, this study first sets *C* to 1, then selects different values of *σ* within the range [0.1, 0.2, 0.3, 0.4, 0.5], and different values of *l* within the range [0.05, 0.1, 0.15, 0.2, 0.25] for network search, recording the validation *R*^2^ values. The experimental results are shown in the heat map, as shown in **Figure 11**. The vertical axis represents different values of *l*, the horizontal axis represents different values of *σ*, and the color bars represent different ranges of model validation *R*^2^ results. The results indicate that the model performance first increases and then decreases with the change in *σ*. When *σ* increases from 0.1 to 0.4, the model *R*^2^ gradually improves, indicating that increasing the standard deviation can make the label distribution more uniform, reduce the model’s dependence on a single data point, thereby reducing the overfitting caused by data noise, and enhancing the model’s generalization ability. However, when *σ* further increases to 0.5 and above, *R*^2^ begins to decrease, which may be due to the label distribution becoming too smooth, causing the model to lose its ability to capture local information, leading to a decrease in prediction accuracy. From the experimental results, *σ* = 0.4 is the optimal choice for this experiment. Regarding the interval length *l* of the label distribution, our results indicate that appropriately increasing *l* can enhance model performance. When *l* increases from 0.05 to 0.15, the *R*^2^ value gradually improves, suggesting that moderately increasing the dispersion interval can reduce the fluctuation in predicted values, thereby making the model regression more robust. However, when *l* continues to increase to 0.2, the model performance declines. The possible reason is that an excessively wide interval leads to a decrease in predictive resolution, making it difficult for the model to accurately capture the subtle changes in Ψ_leaf_. Based on the experimental results, *l* = 0.15 is considered the optimal choice as it strikes a balance between enhancing predictive accuracy and avoiding excessively high computational complexity. In the heat map **Figure 11**, it can be observed that the combination of *σ* = 0.4 and *l* = 0.15 yields the most intense red color, indicating that this is the optimal hyperparameter set for the model, providing the best predictive performance. When *σ* = 0.4 and *l* = 0.15, the model achieves the highest average validation *R*^2^ value of 0.9395, which demonstrates the importance of appropriate label smoothing and reasonable interval division in predictive modeling.

With the standard deviation *σ* of the distribution of the regression network and the interval length *l* set as the optimal values of 0.4 and 0.15, respectively, this study further explores the impact of the hyperparameter *C* in the calculation of the linear shrinkage weight on model performance. The hyperparameter *C* determines the weight assigned to low-frequency data in the KL divergence calculation. A value that is too small may lead to an overemphasis on low-frequency data, thereby affecting the learning of high-frequency data, while a value that is too large may suppress the influence of low-frequency data on model optimization. Therefore, this research conducted experiments with *C* values ranging from [0, 1, 2, 3, 4, 5, 6] and recorded the corresponding validation *R*^2^ values.

**Figure 12:**
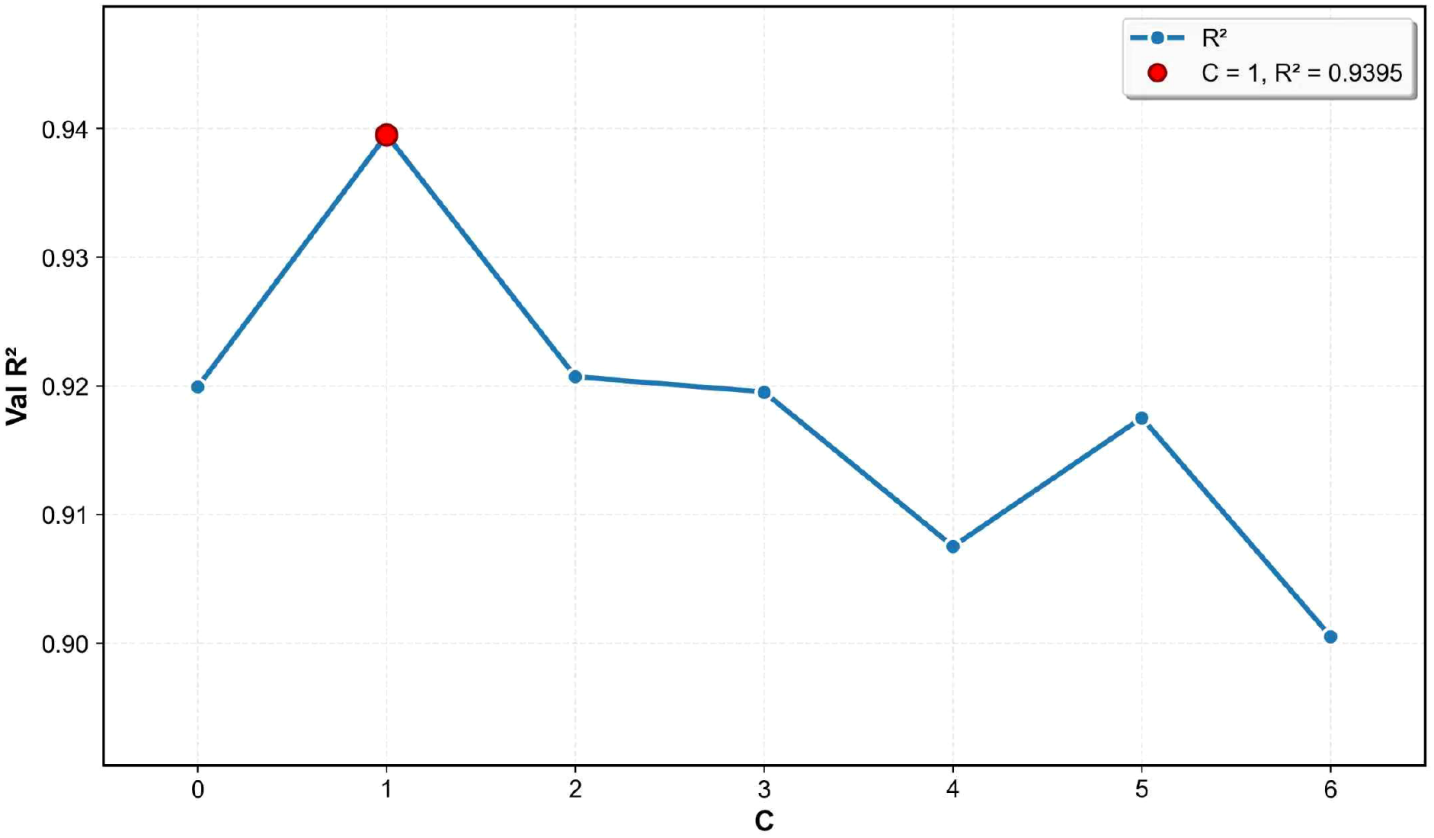
Variation in the validation set *R*^2^ with different values of the regression loss weight coefficient (*C*), which balances the proportion between point-based regression and label distribution learning. This parameter indirectly modulates the model’s sensitivity to low-frequency samples. Experimental results demonstrate that the model performance reaches its peak at *C* = 1.

The experimental results, as shown in **Figure 12**, indicate that as *C* gradually increases from 0 to 1, the model performance improves, with *R*^2^ rising from approximately 0.92 to 0.9395, suggesting that an appropriate *C* value can effectively balance the influence of low-frequency data and high-frequency data during model optimization, enhancing predictive stability. When *C* = 1, *R*^2^ = 0.9395 reaches its maximum value, indicating that at this point, the model can optimally consider the impact of data imbalance while ensuring overall fitting accuracy. As *C* continues to increase beyond 5, *R*^2^ decreases, which may be due to the larger *C* values diminishing the influence of low-frequency data, leading to a reduction in predictive accuracy for data imbalance. Ultimately, this study selects *C* = 1 as the optimal hyperparameter. The results further verify the role of reasonable KL divergence weight calculation in enhancing model predictive capabilities, providing an optimal parameter setting scheme for Ψ_leaf_ prediction tasks. 

## 4 Conclusions

This study presents a deep learning-based regression method for the prediction of leaf water potential from hyperspectral data, named CIDL, which combines CGAN for data augmentation, IRAC for feature extraction, and DARN for optimizing the regression process. Our results demonstrate that CIDL achieved a test *R*^2^ of 0.78, clearly outperforming traditional machine learning methods (mean *R*^2^ = 0.66) and providing a modest yet consistent improvement over mainstream deep learning approaches (mean *R*^2^ = 0.76). Although built upon the already high-performing baseline (*R*^2^ ≈ 0.73), our CIDL architecture provided an absolute improvement of ∼ 0.06 in *R*^2^, equivalent to roughly a 7.63% relative gain. Furthermore, the ablation study and hyperparameter optimization analysis confirm the contribution of each module of CIDL to model performance and determine the optimal hyperparameter combination, further enhancing the model’s stability and robustness. The research indicates the potential of deep learning methods in modeling complex spectral data, especially after combining data augmentation, feature extraction optimization, and label distribution regression strategies, which can effectively improve Ψ_leaf_ prediction accuracy.

Despite these encouraging results, several limitations should be acknowledged. First, hyperspectral imaging is subject to multiple sources of uncertainty, including leaf curvature effects and illumination inconsistency, that were not explicitly quantified here but may compromise the model’s predictive robustness in practical deployment. Second, this model was developed and validated using only 229 direct Ψ_leaf_ and hyperspectral measurements on a single tree species collected from controlled conditions. This limitation may constrain the model’s generalization capacity and hinder its direct application to UAV- or satellite-based platforms, which must account for greater biological and environmental variability (e.g., canopy structural complexity, BRDF anisotropy, background reflectance contamination, etc.) and platform-specific sources of uncertainty (e.g., coarse spatial resolution, motion blur). Future work should focus on transferring the lab-trained model to field scenarios by freezing the feature extraction layers and progressively fine-tuning only the fully connected layers using small fractions of field-collected Ψ_leaf_ data (e.g., 5–20% labeled samples), thereby combining robust spectral features from controlled experiments with efficient, domain-specific calibration from UAV or satellite campaigns. This approach may bridge the lab-to-field gap, laying the foundation for scalable, smart forestry systems that monitor forest water status across landscapes.

## Data Availability

The raw data supporting the conclusions of this article will be made available by the authors, without undue reservation.
