# Supplementary material for: A deep learning architecture for leaf water potential prediction in Populus euramericana ‘I-214’ from hyperspectral reflectance
Source: Front Plant Sci. 2026 Jan 26;16:1709473. doi: 10.3389/fpls.2025.1709473 (PMC12883839; doi:10.3389/fpls.2025.1709473)
Supplement: Supplementary file 1 [file Table1.docx]

**Supplementary Materials:**

**A [Deep Learning](https://www.sciencedirect.com/science/article/pii/S0168169923004222) [Architecture](https://www.nature.com/articles/s42256-021-00309-y) [for Leaf Water Potential Prediction in](https://www.sciencedirect.com/science/article/pii/S0168169923004222) *[Populus euramericana](https://www.sciencedirect.com/science/article/pii/S0168169923004222)* [‘I-214’](https://www.sciencedirect.com/science/article/pii/S0168169923004222) from hyperspectral reflectance**

Xue-Wei Gong^1^, Qing-Song Yu^2^, Hong-Li Li^2^, Zhuo-Qun Fang^2^, Jia-Xu Guo^2^, Zhao-Kui Li^2*^, Heng-Fang Wang^3^, Zhong-Yi Pang^4^, Yan-Hui Peng^4^, Xue-Kai Sun^1^, Guang-You Hao^1*^

^1^CAS Key Laboratory of Forest Ecology and Silviculture, Institute of Applied Ecology, Chinese Academy of Sciences, Shenyang 110016, China;

^2^School of Computer Science, Shenyang Aerospace University, Shenyang 110136, China;

^3^Key Laboratory of Oasis Ecology of Education Ministry, College of Ecology and Environment, Xinjiang University, Urumqi 830017, China;

^4^State-owned Xinmin City Mechanical Forest Farm, Shenyang 110300, China.

^*^Corresponding author:

Zhao-Kui Li^2*^, Guang-You Hao^1*^

(lzk@sau.edu.cn; [haogy@iae.ac.cn](mailto:haogy@iae.ac.cn))

**Table S1** Comparisons of training and testing times between the baseline model and the CIAL method proposed in the present study.

| Models | training time/s | testing time/s |
| --- | --- | --- |
| Baseline | 5.2678±0.7891 | 0.0061±0.0024 |
| CIAL | 29.3264±1.0871 | 0.0060±0.0032 |

|  |
| --- |

**Table S2** Performance comparison of all models on test data. Different letters following the values indicate significant differences in the performance metric among models (*P* < 0.05, HSD post-hoc test following one-way ANOVA). Test *R*^2^, coefficient of determination on the test set; RMSEP, root mean square error of prediction; SDR, standard deviation ratio; MAE, mean absolute error; RPD, ratio of prediction deviation; RPIQ, ratio of performance to interquartile distance.

| Models | Test *R*^2^ | RMSEP (bar) | SDR | MAE (bar) | RPD | RPIQ |
| --- | --- | --- | --- | --- | --- | --- |
| PLSR | 0.7310±0.0716c | 3.0019±0.3699bc | 1.9919±0.2670c | 2.3794±0.3056b | 1.9968±0.2661c | 3.0414±0.4895c |
| SVR | 0.5914±0.0872d | 3.7099±0.4281c | 1.6130±0.2121d | 2.7692±0.2875c | 1.6130±0.2121d | 2.4697±0.4952d |
| SpectraNet32 | 0.7793±0.0525ab | 2.7249±0.3092a | 1.9272±0.2820c | 2.2178±0.2712a | 2.1937±0.2570a | 3.3527±0.5946a |
| DeepSpectra | 0.7520±0.0442bc | 2.9060±0.3481b | 2.0297±0.1803bc | 2.3015±0.2542b | 2.0526±0.1824b | 3.1410±0.5474b |
| CNN | 0.7720±0.0487ab | 2.7717±0.2779b | 2.1285±0.2385b | 2.1680±0.2695a | 2.1526±0.2412b | 3.2887±0.5813b |
| Transformer | 0.7448±0.0483bc | 2.9362±0.2695b | 2.0063±0.2107b | 2.3247±0.2144b | 2.0290±0.2131c | 3.0814±0.3654c |
| CIDL | 0.7842±0.0448a | 2.7095±0.3745a | 2.4965±0.1120a | 2.0848±0.2362a | 2.2108±0.2405a | 3.3835±0.6353a |
